# Behavioral abnormalities are common and severe in patients with distal 22q11.2 microdeletions and microduplications

**DOI:** 10.1002/mgg3.146

**Published:** 2015-04-16

**Authors:** Valerie Lindgren, Anne McRae, Richard Dineen, Alexandria Saulsberry, George Hoganson, Michael Schrift

**Affiliations:** 1 Department of Pathology, University of IllinoisChicago, Illinois; 2 Department of Pediatrics, University of IllinoisChicago, Illinois; 3 Department of Psychiatry, University of IllinoisChicago, Illinois

**Keywords:** Distal 22q11.2 microdeletion, distal 22q11.2 microduplication, low copy repeats, neuropsychiatric problems

## Abstract

We describe six individuals with microdeletions and microduplications in the distal 22q11.2 region detected by microarray. Five of the abnormalities have breakpoints in the low-copy repeats (LCR) in this region and one patient has an atypical rearrangement. Two of the six patients with abnormalities in the region between LCR22 D–E have hearing loss, which has previously been reported only once in association with these abnormalities. We especially note the behavioral/neuropsychiatric problems, including the severity and early onset, in patients with distal 22q11.2 rearrangements. Our patients add to the genotype–phenotype correlations which are still being generated for these chromosomal anomalies.

## Introduction

The now common clinical use of microarray analysis has allowed the identification of many small recurring deletions and duplications (Emanuel and Saitta 2007), often arising through nonallelic homologous recombination (NAHR) at low-copy repeats (LCRs) (Shaikh et al. 2007). The most commonly occurring microdeletion is proximal microdeletion 22q11.2 or DiGeorge/velocardiofacial syndrome, present in about 1 in 2000–6000 live births (Wilson et al. 1994). The frequency of the reciprocal microduplication is unclear at present (Tucker et al. 2013). Both the duplication and the deletion are facilitated by LCRs in 22q11.2, labeled LCR22 A–D from centromere to telomere (alternatively the LCRs are numbered). Rearrangements of 1.5 Mb or more often 3 Mb in size are typically generated through NAHR between the proximal repeats LCR22 A and B, and A and D, respectively. The phenotypic features of these two abnormalities are relatively well documented and are the subjects of comprehensive reviews (Firth 2009; McDonald-McGinn et al. 1999).

More recently the occurrence of microdeletions and microduplications in the distal portion of chromosome band 22q11.2, distinct from DiGeorge/velocardiofacial syndrome, has been appreciated (Ben-Shachar et al. 2008; Coppinger et al. 2009). These abnormalities are typically generated by NAHR involving the more distal LCRs D through H. Previously, the distal abnormalities have been lumped together and referred to as distal 22q11.2 microdeletions (OMIM #611867) and microduplications. However, it is becoming clear that the abnormalities of distal 22q11.2 are more variable in size than those of the proximal region and the phenotypes, also variable, have not yet been well characterized. Microduplications of both proximal and distal portions of the band are more commonly inherited and often milder than the deletions (Coppinger et al. 2009; Ou et al. 2008; Wincent et al. 2010). Consequently, more reports of individuals with deletions have been published (summarized in Fagerberg et al. 2013; Mikhail et al. 2014; Carvalho et al. 2014). Detailed information regarding the phenotypic range of both deletions and duplications is essential in order to counsel and provide appropriate surveillance and management for patients and their families.

We present six individuals, three with the relatively common distal deletion of the D–E region as well as three with duplications, one of D through an atypical breakpoint not involving a LCR and two relatively uncommon duplications of E–H and of F–H. Two of these patients with abnormalities of distal 22q11.2 involving a breakpoint in LCR22 D have hearing loss. Behavior and psychiatric problems are strikingly prevalent in our patients and constitute a major reason they were brought to medical attention.

## Materials and Methods

### Patients

Three (1, 4, 5) of the patients were examined in UIC Pediatric Genetics clinic by GH; 2 patients (2, 3) are sisters originally seen in Developmental Pediatrics Clinic by AS and subsequently by another geneticist; patient 6 was a psychiatric inpatient cared for by MS. Only one of the parents of these individuals has been tested for the chromosome abnormalities detected in their children, the mother of patients 2 and 3 who was negative for the deletion.

Patient 1, a female, first presented at 15 months of age with duodenal stenosis/atresia, motor delay, hypotonia, poor feeding, pre- and postnatal growth restriction and microcephaly. The duodenal stenosis/atresia is believed to be contributory to her postnatal growth failure, as subsequent to surgical intervention her growth rate has improved somewhat; however, she remains below the 2nd percentile. Her mother’s pregnancy was complicated by preeclampsia necessitating delivery at 36 + 6 weeks of gestation. Her motor development is delayed likely secondary to her persistent hypotonia. She was last assessed at our institution at the age of 21 months, at which time she was not walking and was receiving physical therapy for hypotonia. She also had strabismus. Intellectual ability awaits assessment due to her young age at the time of last evaluation by the genetics service. Unfortunately, though she is now over 3 years old, she has failed to return for follow-up appointments.

Patient 2, a 5-year-old female, presented to Developmental and Behavioral Pediatrics with sensorineural hearing loss in both ears and possible auditory neuropathy spectrum disorder of the left ear. She was delivered at 39 + 4 weeks following uncomplicated labor and delivery. Growth parameters were appropriate for gestational age. There was neither history of ear infections nor a family history of hearing loss. She has eczema. Her speech is delayed and difficult to understand. The patient’s cognitive abilities have been difficult to test due to the hearing and behavioral problems but she is judged to be behind her peers in academic skills. Due to symptoms of mood lability, self-harm, and aggressive behaviors, she was previously admitted to a psychiatric hospital, where she was diagnosed with attention deficit/hyperactivity disorder (ADHD) and pediatric bipolar disorder.

Patient 3 is the younger sister of patient 2 and first presented to Developmental and Behavioral Pediatrics at 4.75 years of age. She was delivered at 34 + 1 weeks due to severe preeclampsia and intrauterine grow retardation. Birth weight was 1605 g. She has bilateral syndactyly of the second and third toes, microcephaly, possible submucosal cleft palate, and eczema. She has had repeated doctor visits and hospitalizations for moderate persistent asthma. Articulation errors have been noted in her speech. On the Bracken School Readiness Test (Bracken 2007), a developmental test that evaluates foundational academic concepts, she was found to be at the third percentile. On the Beery-Buktenica Visual-Motor Integration Test (Beery et al. 2010), a developmental test that evaluates visual perception and motor integration, she performed at the 21st percentile. She has ADHD, oppositional defiant disorder and other behavior problems, including pica. She was recently hospitalized for violent acts toward a sibling.

Mental illness is reported in the paternal grandfather and three paternal uncles of sisters 2 and 3. Their father reportedly has bipolar disorder and received special education. Their mother has been tested and does not have the deletion; given the strong family history of mental illness in the father and the presence of the deletion in two sisters, it is possible that he carries the deletion, although he has not been tested.

Patient 4 was referred to genetics clinic at 8 years of age after a diagnosis of bilateral sensorineural hearing loss at least partially remediated with hearing aids and speech therapy. His mother’s pregnancy was unremarkable with a vaginal delivery at term. He has relative macrocephaly with underdeveloped helices and an epidermal nevus on the top of the head and cowlick. His height and weight are at the 3–5th percentile. His medical history was negative for recurrent ear infections. He had a single seizure at 2 years of age, but has not suffered any subsequent ones. He also has myopia and strabismus, with significant history of myopia on both sides of the family. His paternal grandfather and uncle both have a history of seizures. His father has had hearing loss since childhood. The child is currently on medication for ADHD.

Patient 5 is a 17-year-old female with intellectual disabilities. Her growth parameters are within the normal range except for weight which is greater than the 99 percentile. She has asthma, dyslipidemia and elevated A1C. She compulsively picks her skin. She has no facial dysmorphism. Her family history of intellectual deficits is extensive: her father, one sister, and two brothers also have intellectual disabilities to varying degrees, with her brothers reportedly being more severely impaired. She also has a sister with normal intelligence who has difficulty with anger management. This patient’s two brothers tested negative for the duplication; her sisters have not had genomic testing. The patient is in foster care and recently delivered a daughter who has not yet been evaluated for the duplication.

Patient 6 is a 21-year-old male with intellectual disabilities and a seizure disorder. He has no facial dysmorphism. His development had been normal: he learned to walk at less than one year of age, was toilet trained at 16 months and spoke in sentences at 2 years. His first seizure occurred at 5 years 10 months and was described as a generalized seizure. However, the EEG study suggests a focal onset with left temporal sharp waves. His CT scans and MRIs of the head have been normal. His mother has had seizures since the age of 16 that are generalized. In addition, a paternal great-aunt has multiple congenital anomalies (not specified) and epilepsy and a paternal great-grandfather also had epilepsy. The patient has a flat affect and has received special education. He has had three psychiatric inpatient admissions for drastic changes in behavior—including talking to himself, writing on himself, refusing to shower, refusing to go outside, sleeping in the closet or at the foot of his bed, not sleeping well, wanting to be fed, and being withdrawn, as well as needing 24 h supervision. He has had past diagnoses of ADHD, bipolar disorder, and psychosis. He is currently taking antiepileptic, antidepressant, and antipsychotic medications.

### FISH analysis

All unique abnormalities detected by array analysis were confirmed by FISH analysis using cosmid probes [obtained from Signature Genomics (Spokane, WA), listed in Table1] that were labeled with the Abbott Molecular (Des Plaines, IL) nick translation labeling kit. FISH followed the Abbott Molecular protocol for single copy sequences.

**Table 1:** Clinical and molecular characteristics of 6 patients with distal 22q11.2 abnormalities.

	Patient 1	Patient 2	Patient 3	Patient 4	Patient 5	Patient 6
Age at evaluation (year)	1.33	5	4.67	8.25	17	21
Gender	Female	Female	Female	Male	Female	Male
LCR deletion interval	D–E	D–E	D–E	D–atypical between D and E	F–H	E–H
Minimum size of deletion/duplication	1.16 Mb loss	1.16 Mb loss	1.16 Mb loss	432 Kb gain	1.1 Mb gain	1.94 Mb gain
Chr 22 coordinates [hg 19]	21798705–22962918	21798705–22962918	21798705–22962918	21798705–22230422	23891773–24991691	23055147–24991691
FISH probe confirmation	RP11-647D11×1	CTD-2505D8×1	Not done	RP11–647D11×3	RP11–1087B15×3	RP11–1087B15×3
Family status	Lives with parents	Lives with father, mother, and sibs, 4 sibs with ADHD, mental illness in four paternal relatives, schizophrenia and developmental delays in paternal relatives	Younger sister of patient 2	Paternal grandfather and uncle with seizures, myopia on both sides of family, father with childhood hearing loss	In foster care, strong family history of intellectual disability, daughter not yet tested for duplication	Lives with mother
Birth	36+6 week, preeclampsia	39+4 week, no complications	34+1 week, preeclampsia, IUGR, reduced fetal movement	Term, vaginal	Unknown	Unknown
Birth weight	1764 g	3288 g	1605 g	3373 g	Unknown	Unknown
Weight centile	0.08	<2	39	3–5	99	Unknown
Height centile	6	80	63	3–5	50	Unknown
Head circumference centile	0.9	75	<2nd percentile	50	25	Unknown
Facial anomaly	–	Long face, micrognathia	Long face, tubular nose, malar hypoplasia, long smooth philtrum, thin upper lip, micrognathia, high arched palate, submucosal cleft	Underdeveloped ear helices, facial nevus	–	–
Heart disease	–	–	–	–	–	–
Palatal abnormality	–	–	Submucosal cleft palate	–	–	–
Recurrent infections	–	–	–	–	–	Unknown
Feeding problems	Poor feeding, emesis, likely due to duodenal stenosis/atresia	–	–	–	Unknown	Unknown
Hearing loss	–	Sensorineural loss with auditory neuropathy spectrum disorder (ANSD) in one ear	Evaluation in process	Bilateral sensorineural, wears hearing aids	–	–
Eye abnormality	Strabismus	–	–	Myopia, strabismus	Myopia	–
Neurological abnormality	Hypotonia, failure to thrive	ADHD, pediatric bipolar disorder, aggressive behavior, good muscle tone	ADHD by DSM-V Oppositional defiant disorder/conduct disorder, aggressive behavior	1 seizure at 2 year, paternal family history of seizures and hearing loss, ADHD, CT, and MRI normal	–	Seizures, epilepsy, psychoses, anxiety, ASD with intermittent explosive disorder
Urinary tract anomaly	–	–	–	Bed wetting	–	–
Gastrointestinal anomaly	Duodenal stenosis/atresia	–	–	Chronic constipation related to rectal muscle development, now normal	Obesity	–
Developmental delay	Motor delay	Speech and language, speech often unintelligible	Speech and language, unintelligible speech, BSRA-3: 3rd percentile; Beery VMI: 21st percentile, mildly delayed gross motor milestones	Speech therapy	Unknown	–
Intellectual disability	Too young to evaluate	Learning disorder, IEP	IEP in place	Unknown	+, special education	Mild, special education
Other	–	Head banging, aggressive, and destructive behavior, eczema, deletion not maternally inherited,	Bilateral syndactyly 2-3 toes, severe asthma, eats inedible objects, eczema, aggressive behavior, self -injurious behavior	Relative macrocephaly	Asthma, dyslipidemia, elevated A1C, skin picking, father had special education	

ADHD, attention deficit with hyperactivity disorder; ASD, autism spectrum disorder; Beery VMI, Beery–Buktenica Developmental Test of Visual-Motor Integration, 6th edition; BRSA-3, Bracken School Readiness Assessment Test, 3rd edition; IEP, individualized educational program; IUGR, intrauterine growth retardation.

### Microarray analysis

DNA was manually extracted using the Qiagen (Germantown, MD) Puregene Gentra blood kit. Hybridization was performed using Agilent 4 × 180K oligonucleotide arrays designed by Signature Genomics, according to the manufacturer’s instructions. Slides were scanned using a Roche (Indianapolis, IN) MS200 scanner and data were analyzed with Agilent (Santa Clara, CA) CytoGenomics 2.0 software, then displayed using the Signature Genomics Genoglyphix® software. All abnormalities are described using human genome build 19.

## Results

**Figure 1:**
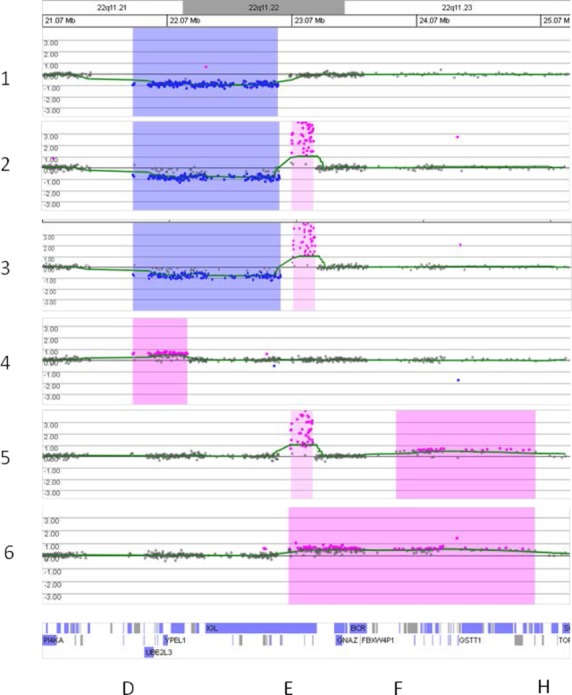
Array comparative genomic hybridization analysis plots of log_2_(patient/reference signals) on the vertical scale versus position of the oligonucleotide clone on chromosome 22 in the region of q11.21–q11.23 on the horizontal scale. Analysis of patients 1–6 is shown from top to bottom. Blue shading indicates a loss in the patient compared to the control while pink indicates a gain. The UCSC Genome Browser position of genes is below the sixth plot and the letters D, E, F, and H at the bottom represent the approximate locations of the low copy repeat (LCR) sequences LCR22 D–H. Patients 1, 2, and 3 have deletions generated by rearrangements involving LCRs D and E, while patients 5 and 6 have duplications flanked by LCR22 H on the distal end and LCR22s E and F, respectively, on the proximal end. Patient 4 has an atypical duplication generated using LCR22 D on the proximal end but the distal breakpoint is not at a LCR. The areas shaded with lighter pink for patients 2, 3, and 5 represent benign variant gains within the *IGL* gene.

Microarray analysis demonstrated abnormalities of distal 22q11.2 in each of our six patients; the results for chromosome 22 are shown in Figure1. Patients 1, 2, and 3 have deletions that extend from LCR22 D through LCR22 E. The proximal portion of the LCR22 D–E interval comprises the duplication of patient 4 but the distal breakpoint is between LCRs D and E. Patient 5 has a duplication generated using LCR22 F and LCR22 H and patient 6 has a larger duplication from LCR22 E–H. Since only one of the parents of these individuals has been tested, the inheritance patterns of the abnormalities are not established. Table1 summarizes the clinical findings in each patient.

## Discussion

Microdeletion and microduplication of 22q11.2 in the DiGeorge/velocardiofacial region are very common genomic abnormalities that occur through NAHR of the four proximal LCRs A–D present in proximal chromosome 22q11.2. However, abnormalities in 22q11.2 but distal to the DiGeorge/velocardiofacial region and generated using the 5 distal repeats D–H are increasingly being reported. A phenotype is emerging for deletion of the D–E segment (Carvalho et al. 2014; Mikhail et al. 2014), but other abnormalities are relatively rare and information is lacking.

We have described six new patients, three with microdeletions and three with microduplications, of distal 22q11.2. Five of the rearrangements were facilitated by the LCR 22s D–H. The duplication of patient 4 used LCR D as the proximal breakpoint and an atypical distal breakpoint between LCRs D and E just telomeric to the *PRAME* gene (OMIM gene #606021). A single patient described by Xu et al. (2008) has similar breakpoints but has a deletion. One other patient with a similar duplication has also been reported (Wincent, 2010). Therefore, there may be sequences in this region that facilitate rearrangements and additional cases may be uncovered in the future. In line with the observations of others (Shaikh et al. 2007; Coppinger et al. 2009; Wincent et al. 2010), we did not observe any rearrangements involving LCR G which has the opposite orientation to LCRs E, F, and H.

The deletions involving LCRs D–E appear to be the most common with at least 26 patients previously reported (Saitta et al. 1999; Ben-Shachar et al. 2008; Newbern et al. 2008; Rødningen et al. 2008; Xu et al. 2008; Bruce et al. 2010; Tan et al. 2011; Verhoeven et al. 2011; Fagerberg et al. 2013; Molck et al. 2013; Mikhail et al. 2014; Carvalho et al. 2014). Carvalho et al. (2014) have presented a recent literature review of all patients with this deletion with the exception of the six cases reported by Mikhail et al. (2014). The most prominent features include prematurity, pre- and postnatal growth restrictions; microcephaly; smooth philtrum; hypoplastic nasal alae; pointed chin and nose; posteriorly rotated ears; congenital heart defects; developmental delay, particularly affecting speech; intellectual disability; and behavior problems. Our patients 1 and 3 demonstrated pre- and postnatal growth restriction; patient 2 with the same deletion has postnatal growth restriction. The *MAPK1* gene (OMIM gene #176948) encoding mitogen-activated protein kinase 1 which is within the D–E region is a candidate gene for this feature of the distal deletion 22q11.2 phenotype (Mikhail et al. 2014). In contrast, the growth of patient 4 with duplication of part of this region including *MAPK1* is within normal parameters with relative macrocephaly. Patients 1 and 3 were delivered preterm due to preeclampsia in the mothers. This has been a relatively common finding in patients with this genomic abnormality, as noted by Mikhail et al. (2014) and points to the necessity for awareness of this feature in any cases detected prenatally. Also conforming to the findings in other patients with this deletion, patients 1, 2, and 3 have speech and language delays although hearing loss is a confounding factor in the assessment of patient 2.

Patient 2 with a deletion and patient 4 with a duplication have sensorineural hearing loss. Patient 2 was initially found to have cerumen impaction but upon its removal sensorineural loss was diagnosed. The abnormality in both patients involves LCR22 D as the proximal breakpoint but the deletion of patient 2 extends to LCR22 E while the duplication of patient 4 has a breakpoint between LCR22 D and LCR22 E. This is only the second report of hearing loss associated with abnormalities in this region: the patient of Rødningen et al. (2008) with sensorineural hearing loss has the same deletion as our patient 2. The sister of patient 2 does not have defined hearing loss, but additional screening is pending. As there is a history of hearing loss in the father of patient 4, another etiology cannot be excluded but disruption of gene expression of a gene in the D–E region is possible.

Duplications involving LCR22 E–H and F–H have been reported to have a variable phenotype with little correlation with size of the abnormality but features include microcephaly, seizures, hypotonia, developmental delay, and neuropsychiatric issues (Descartes et al. 2008; Ou et al. 2008; Coppinger et al. 2009; Wincent et al. 2010; Shimojima et al. 2010). Neither patient 5 nor 6 had gross motor delay or any distinguishing physical characteristics except obesity in patient 5. Of note, one of this patient’s siblings who tested negative for the duplication is also obese, suggesting a possible unrelated familial susceptibility or environmental factors. Both patients 5 and 6, now young adults, did require special education. Seizures have occurred in two of our three duplication cases, patients 4 and 6, although these duplications do not overlap.

Behavior and psychiatric problems are particularly striking in this group of individuals, especially in the D–E deletion patients. Patient 1 is too young to be assessed for behavior, but patients 2, 3, 4, and 6 have ADHD. Sisters 2 and 3 demonstrate aggressive behaviors, with both requiring hospitalization for threatening behavior and the younger one being expelled from several schools. Patient 2 also has pediatric bipolar disorder and patient 6 is being treated for mood dysregulation and psychosis and has required multiple hospitalizations for behaviors. Patients 3 and 5 have dermatillomania. Another factor that may be influencing behavior but is difficult to assess is the probable presence of some of these genomic rearrangements in parents (especially patients 2 and 3) and resulting unstable family environments.

Behavior abnormalities have been highlighted in the reports of some patients with distal 22q11.2, such as the case of Ribeiro-Bicudo et al. (2013) with an inherited LCR F–H duplication. His behavior was described as ranging from apathy to extreme anxiety with immaturity and dependence on his mother for any decision. Unspecified severe behavior problems were reported in a patient with a D–H duplication (Coppinger et al. 2009). It is difficult to judge the frequency of behavior problems in the group of duplication patients studied by Wincent (2010), as behavior features are grouped with autistic features.

A high proportion of deletion patients have behavior difficulties as well. In the study of Mikhail et al. (2014) four of their six patients with LCR D–E deletions have behavior issues including immaturity, talking to oneself, impulsivity, anger issues, anxiety, ADHD, and Asperger disorder. Behavior problems in other cases with D–E deletion include: a patient of Saitta et al. (1999) was extremely shy; Ben-Shachar et al. (2008) describe aggression in one patient; Verhoeven et al. (2011) reported a patient with panic disorder and anxiety; Tan et al. (2011) had two patients with food issues; Fagerberg et al. (2013) and Molck et al. (2013) each describe a patient with attention deficit and one of the patients of Fagerberg et al. (2013) has food craving. In all 11 (42%) of the 26 D–E deletion patients previously reported have behavior issues.

In our patients behavior problems are severe and of early onset. Although several of the patients have intellectual disabilities/learning difficulties and physical anomalies such as hearing loss and duodenal stenosis, the behavior issues have been the most common reason for medical attention and hospitalization. In fact, one of our patients (6) came to attention directly from Psychiatry and two of the girls have been hospitalized for aggressive behavior around the age of 4–5. As with deletion 22q11.2 patients who have a propensity to develop psychosis (summarized in Murphy 2002), psychiatrists and neurologists should consider distal 22q11.2 abnormalities as an etiology in their patients, especially in the younger ones.
